# Perspective on coronary interventions & cardiac surgeries in India

**Published:** 2010-11

**Authors:** Upendra Kaul, Vineet Bhatia

**Affiliations:** Escorts Heart Institute, New Delhi, India; * *Fortis Hospital, NOIDA, India*

**Keywords:** Angioplasty, CABG, cardiac cath laboratories, cardiac surgery, cardiovascular disease, coronary intervention, coronary stents

## Abstract

Cardiovascular disease has become the leading cause of morbidity and mortality in India during the last 3 decades. The genetic predisposition and acquisition of traditional risk factors at a rapid rate as a result of urbanization seems to be the major cause. While efforts are being made to contain this epidemic by educating public and applying preventive measures, the ever increasing burden of patients with symptomatic and life threatening manifestations of the disease is posing a major challenge. This requires a concerted effort to develop modern facilities to treat these patients. The healthcare facilities to manage these high risk patients by contemporary methods like percutaneous coronary revascularization and surgical methods have shown a very promising trend during the last decade. The facilities of modern diagnostic methods and new proven techniques to offer symptomatic relief and improve their prognosis are available in most parts of the country. The lack of social security and health insurance for the large majority of the population, however, is a serious limitation. Unregulated availability of some of the newer devices for these techniques had become a very concerning issue. However, in the last few years serious efforts have been made to streamline these procedures. Indigenous research and scientific data acquisition in relation to the modern technology for achieving coronary revascularization has also started on a promising note.

## Coronary artery disease in India-Magnitude of the problem

Studies of Indian immigrants and cross sectional studies in India, have demonstrated that coronary artery disease (CAD) is rampant in Indians and that its prevalence is several folds higher than in industrialized nations. The Global Burden of Diseases (GBD) study^1^ reported the estimated mortality from CAD in India at 1.6 million in the year 2000. Extrapolation of this estimate shows the current burden of CAD in India to be more than 32 million patients. Epidemiological studies show a sizeable burden of CAD in rural (3-5%) and urban (7-10%) populations. A conservative estimate indicates that there could be 30 million CAD patients in India of which 14 million are in urban and 16 million in rural areas^2^. If the current trend continues by the year 2020, the burden of atherothrombotic CVD in India will surpass other regions of the world.

Some peculiarities of CAD patterns stand out in Indian patients. These include: younger age at presentation, a high incidence of double (DVD) and triple vessel disease (TVD), diffuse involvement, distal disease and significant left ventricular dysfunction at presentation. An angiographic study from Vellore^3^ in 1066 consecutive males admitted for CAD noted significant disease in 877 patients; of these, 55 per cent were <50 yr of age, 34 per cent were <45 yr of age and 12 per cent were below 40 yr of age. Although the mean age was 48 yr, TVD was more common (55%) than DVD (24%) and single vessel disease (24%) combined. Reports from New Delhi^4^ have confirmed the high prevalence of TVD. The high prevalence of TVD (35%) was also reported in non-smoking pre-menopausal women^5^. An incidence of TVD in post-menopausal women was reported to be 57 per cent from another center^6^. Another study from north India^7^ evaluated premature CAD. They found that the incidence of angiographically proven CAD in the young (<40 yr) was about 10 per cent. Though these patients had lower prevalence of diabetes and hypertension, smoking and family history of premature CAD was more common in comparison to older patients. Overall, the patient population had lower total cholesterol (TC) and LDL-C levels (as compared to the reported western literature) and high triglyceride (TG) and low HDL-C levels. Younger patients had a more atherogenic lipid profile as reflected by higher TC, LDL-C and TG levels. The angiographic profile also varied with age, with older patients having more diffuse disease and higher frequency of TVD. Amongst predictors of either TVD or sustaining myocardial infarction (MI), diabetes and TC/HDL-C ratio were the strongest predictors. However, these data are from tertiary centers and may reflect referral bias and show only the tip of the iceberg. Thus a large burden of CAD characterized by high severity and poor anatomy exists and threatens the Indian population.

**Fig 1:**
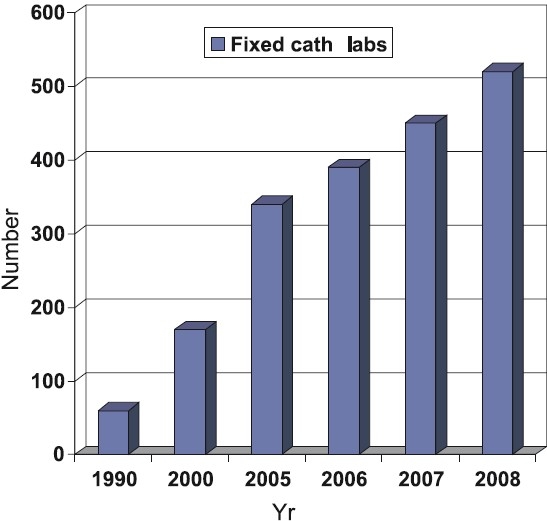
Growth of fixed cath labs in India [*Source*: Philips India, *www.india.philips.com*].

**Fig 2:**
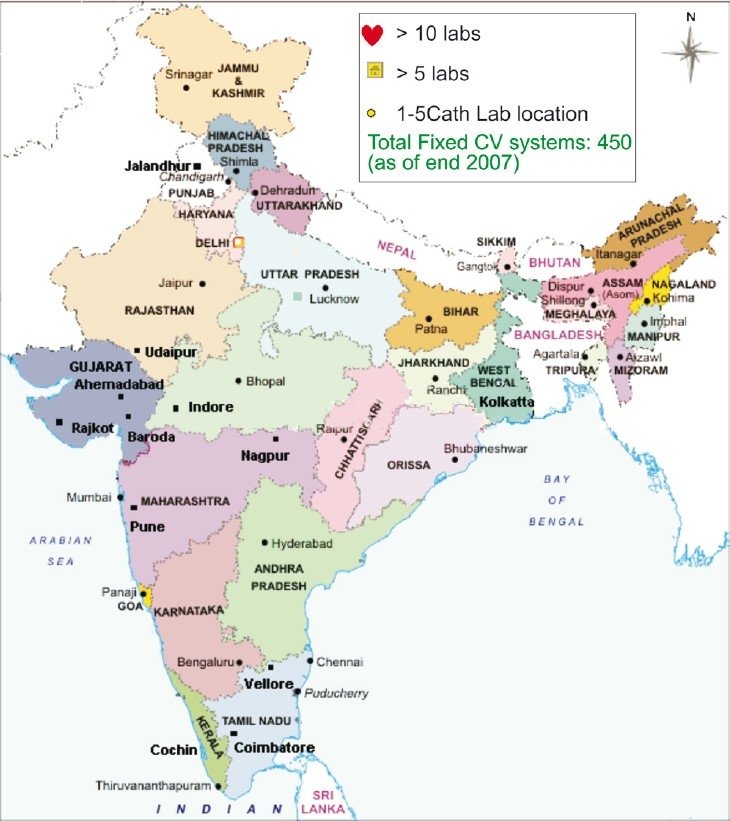
Distribution of cath labs in India [*Source*: Philips India, *www.india.philips.com*].

The mortality attributable to CVD in India is expected to rise by 103 per cent in men and 90 per cent in women from 1985 to 2015^8^. Even as CVD rates skyrocket, the availability of better cardiology facilities and dedicated cardiac centers have come as a welcome relief and India has seen a big leap in the fields of interventional cardiology and cardiac surgery in recent times. There are presently over 500 centers with facilities for coronary angiography and coronary angioplasty in the country^9^ and these numbers are steadily increasing (Fig. 1). Most metropolitan cities have these facilities available and even smaller cities are acquiring these at a rapid rate (Fig. 2). However, high treatment costs are a major hurdle for the economically deprived seeking medical help. They often have to rely on Government hospitals, which are situated in metros and have long waiting lists causing much unwanted delay in treatment. The difficulties encountered during interventional procedures and surgery often relates to late presentation, diffuse disease, small vessel disease and left ventricular (LV) dysfunction. Lack of an organized uniform health insurance policy for these expensive procedures is a major drawback for optimal utilization of these facilities. Patients with conditions like acute coronary syndromes, left main disease, symptomatic multiple vessel disease with left ventricular dysfunction are very often not able to avail these services because of these reasons. Even the long-term optimal medical treatment is availed by a relatively small number of patients because of lack of motivation, long-term cost and availability of good counselling.

## Coronary interventions

**Fig 3:**
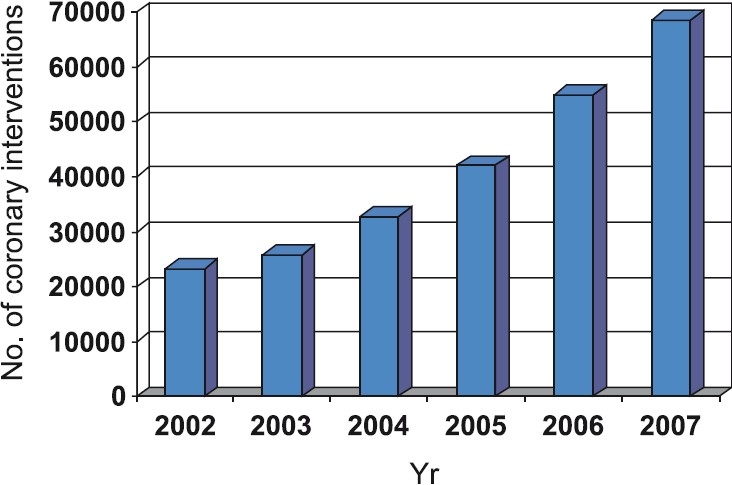
Total coronary interventions in India [*Source*: Ref. 10].

**Fig 4:**
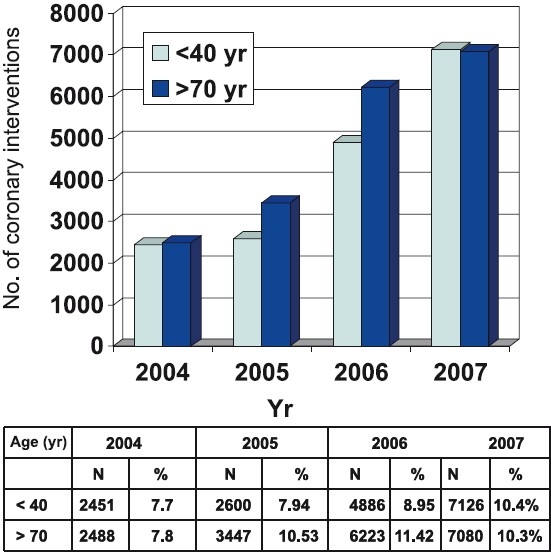
Coronary intervention data as per age group [*Source*: Ref. 10].

**Figure 5:**
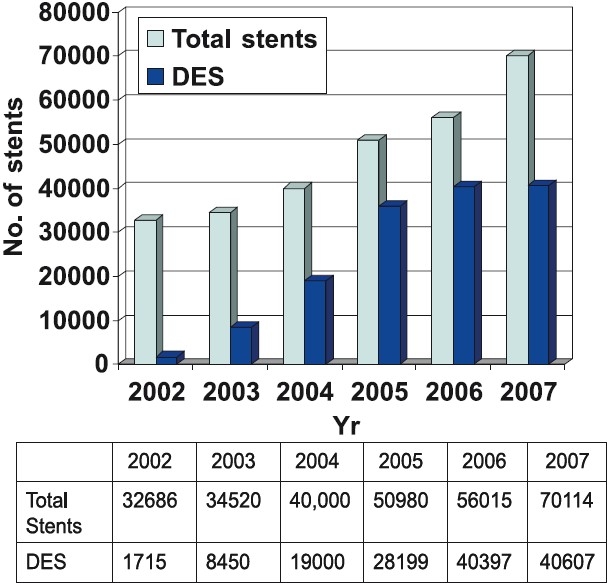
Total number of stents and drug eluting stents (DES) usage patterns in India [*Source*: Ref. 10].

**Figure 6:**
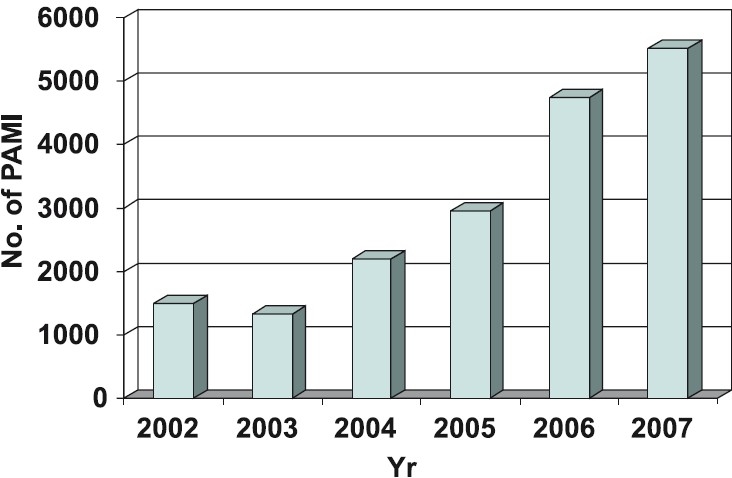
Data on intervention in acute MI [*Source*: Ref. 10].

The data on coronary interventions in India are largely available through two sources - The National Intervention Council (NIC) and the Industry. The NIC has developed a web-based system (*www.nic-csi.org*), which helps individual operators and institutions to upload their data from time to time. There has been a steady annual rise to the tune of 25-30 per cent in the number of interventions over the past several years^10^ (Fig. 3). This on one hand reflects the accessibility of the population to advanced cardiac facilities and on the other hand, portrays that the disease is now achieving epidemic proportions. When patient’s age is considered, it can be seen that septuagenarians are being taken up for angioplasty more readily (Fig. 4). On the other hand, the number of female patients undergoing angioplasty has more or less remained constant at 20–25 per cent of total interventions. The radial approach is also steadily becoming popular in India as is apparent in Fig. 4. Dr Tejas Patel at Ahmedabad, Gujarat, has carried out pioneering work in radial interventions in India having performed over 20,000 transradial procedures. His annual transradial intervention course is one of the most widely attended courses in interventional cardiology and he is the author of atlas of Transradial intervention^11^. The total number of stents used has risen but what is striking is the usage of drug eluting stents (DES). As compared to 2005 where DES constituted 55.13 per cent of total stent usage the numbers in 2006 are a phenomenal 72.11 per cent (Fig. 5). These figures exceed the percentage of DES implantation in some of the industrialized nations of Europe and may be an indicator of rampant overuse in the absence of clear-cut guidelines. A few DESs available in India, which were the issue of a hot debate recently for not having been used even in the country of origin prior to their availability in this country, prompted The Food and Drug Administration (FDA) of India to intervene and set up a panel of experts to reach a consensus. A nation-wide detailed survey of the Indian cardiologists also looked at this issue in great detail^12^. Bodies like the Central Government Health Services and Employees State Insurance have laid down broad guidelines for DES use when it comes to reimbursement issues. The issue of late stent thrombosis after DES usage has influenced stent usage patterns. In a recent study from a leading interventional cardiology center at Fortis Hospital, New Delhi, the DES usage decreased from 68.6 to 63 per cent in the period that followed the European Society of Congress meeting in Barcelona in October 2006^13^. On the other hand, the bare metal stent usage increased from 31 to 37 per cent. The numbers of cases in which other devices such as Rotablater, Intravascular ultrasound (IVUS) and distal protection devices have been used are low. Glycoprotein IIb/IIIa blocker usage has increased and the most commonly used GpIIbIIIa receptor blocker in India is Eptifibatide^9^. Small molecule usage (Eptifibatide and Tirofiban) has increased due to low cost, easy availability, relatively satisfactory safety profile and several local Indian brands being available. The reported complication rates have been rather low and seem a bit unrealistic. The approaches used in case of sub acute stent thrombosis (SAT) are summarized in the Table. In acute myocardial infarction settings thrombolysis continues to be vogue in smaller cities where facilities for primary angioplasty in myocardial infarction (PAMI) are not available and transport facilities do not exist. Patients more than often present late after the onset of symptoms such that the desired goals namely, door to needle and door to balloon times as specified in guidelines are rarely met. In the CREATE registry only 41.6 per cent patients with ST elevation myocardial infarction (STEMI) presented with 4 h of the onset of chest pain 31 per cent patients presented after 12 h^14^. Though agents such as tenecteplase are now available in the Indian market, the concept of pre-hospital thrombolysis is still a distant dream as the ambulance facilities are poor and majority of the times those available are ill equipped. Recently a not for profit organization in public – private partnership known as the Emergency Management Research Institute (EMRI), has established a professional Emergency Services network in several States of India (*www.emri.in*). EMRI handles medical, police and fire emergencies through the “1-0-8 Emergency service”. This free service delivered via state-of-art emergency call response centres and has over 1110 ambulances across Andhra Pradesh, Gujarat, Uttarakhand, Goa, Tamil Nadu and Rajasthan. With the expansion of fleet and services set to spread across more States, EMRI will have more than 10000 ambulances covering over a billion population by 2010. Initiatives like these could help offer timely treatment to patients of acute myocardial infarction such as pre-hospital thrombolysis and help achieve the desired door to needle and door to balloon time goals. Several hospitals in metro cities offer angioplasty services on a 24 × 7 basis at a premium cost. The number of PAMIs has increased in the recent years in India (Fig. 6). Interventions on the left main and graft angioplasty rates in India are low. LMCA Angioplasty in 2006 constituted 0.74 per cent of all interventions and in 2007 remained nearly unchanged at 0.64 per cent^9^. Similarly graft angioplasty rates in 2006 and 2007 were 1.35 and 1.34 per cent respectively of the total procedure number^9^. There has also been a steady rise in the number of fixed and mobile catheterization laboratories in India. The total number of fixed cath labs in 2007 was estimated at about 450 (Fig. 1). The numbers of labs however, are more in the western and southern parts of India with at least 6 cities having more than 10 labs compared to only 3 cities having more than 10 labs in the northern and eastern States (Fig. 2).

As regards to advance training facilities for doctors in interventional cardiology after general cardiology grooming there are only a few organized programmes like post doctoral fellowships restricted to a few centers recognized by Medical Council of India (MCI) or National Board of Examinations (NBE). There is a lot that can be done to train more doctors in cardiology if there is a political willingness for the same. Higher health budget allocations are required to enhance training facilities. The MCI and NBE need to work in unison with the Ministry of Health to establish long-term plans.

## Cardiac Surgeries

Coronary artery bypass graft surgery (CABG) was first performed in India in 1975 about 13 years after its advent in 1962^15^. In the mid 1990 some 10,000 CABG surgeries were being performed annually in India. Presently the annual number is about 60000 according to industry sources. In the absence of a central registry the exact numbers may not be apparent. There are several technical challenges, which cardiac surgeons in India have to face. These are chiefly related to small coronary vessels, arterial conduits, diffuse disease and late presentation^16^. Kinare & Kulkarni^17^ in a smaller series from Mumbai reported that heart weight in Indians varied from 148 to 249 g while in the West the average weight of the heart, in males is 300 g and that in females is 250 g. Such smaller sized vessels pose difficulty during anastomosis and may result in early graft closure leading to higher mortality^16^. The adequacy of the size of the arterial conduits has also been a matter of concern, but autopsy studies by Reddy *et al*^18^ have shown that size of the arterial conduits appears to be adequate to permit their frequent use in CABG. Indians also tend to have diffuse CAD because of which (*i*) vessels frequently require endarterectomy, (*ii*) perioperative myocardial infarction is more likely, and (*iii*) bypass grafts are more likely to occlude after successful surgery. Two Indian studies have reported the requirement of endarterectomy as 14 and 16 per cent in patients with diffuse disease undergoing CABG^19^,^20^. Trehan *et al*^16^ have also reported the presence of mild to moderate inflammation in the coronary arterial walls of some patients undergoing CABG. These areas are patchy areas of erythema on the vessel surface. The atherosclerotic plaque is quite adherent and it is difficult to dissect in this area. Left ventricular dysfunction is also common in Indian patients. In a study from Escorts Heart Institute and Research Center (EHIRC), New Delhi, 20 per cent patients had severe LV systolic dysfunction at time of CABG^15^. In contrast, at Cleveland clinic only 8.5 per cent patients had severe LV dysfunction at surgery. LV dysfunction has a vital bearing on post-operative recovery^16^. Some techniques which have gained popularity in India are off-pump coronary artery bypass grafting (OPCAB), use of minimally invasive techniques (MIDCAB) and robotic surgery or totally endoscopic coronary artery bypass surgery (TECAB). In India, approximately 60 per cent surgeries are off-pump. Some major centers as EHIRC carry out 98 per cent of their cases as OPCAB. The same center pioneered the TECAB introducing it at their center in 2002.Untill 2005, 13 patients were subject to TECAB using the da Vinci with excellent results^21^.

## Conclusions and future direction

Coronary artery disease has reached an epidemic proportions in South East Asia especially India in the recent few years. Unhealthy dietary habits, sedentary lifestyle, atherogenic dyslipidaemia and high incidence of smoking and diabetes have been largely contributory. At the same time however, better medical facilities have come up and coronary interventions and cardiac surgeries are being performed in nearly all major Indian cities, especially the metros. Growing expertise coupled with greater awareness and better affordability amongst patients will have a positive impact in the future. Availability of newer techniques such as new generation DES, OP CAB, MID CAB and TECAB are bound to improve short- and long-term results.
